# Digestibility of Energy and Concentrations of Digestible and Metabolizable Energy in Pistachio Shell Powder and in Soybean Hulls Fed to Growing Pigs

**DOI:** 10.3390/ani16050758

**Published:** 2026-03-01

**Authors:** Yeonwoo Kim, Maryane S. F. Oliveira, Su A Lee, Hans H. Stein

**Affiliations:** Department of Animal Sciences, University of Illinois at Urbana-Champaign, Urbana, IL 61801, USA; yeonwoo3@illinois.edu (Y.K.); maryane.oliveira@dsm-firmenich.com (M.S.F.O.); sualee2@illinois.edu (S.A.L.)

**Keywords:** digestibility, energy, fiber, growing pigs, pistachio shell powder, soybean hulls

## Abstract

The hypothesis that the values obtained for the apparent total tract digestibility (ATTD) of energy and digestible energy (DE) and metabolizable energy (ME) in pistachio shell powder are not different from the values obtained for soybean hulls when fed to growing pigs. A basal diet containing corn, a diet containing corn and 20% pistachio shell powder, and a diet containing corn and 20% soybean hulls were formulated. Twenty-four growing pigs (initial body weight: 32.0 ± 1.7 kg) were allotted to the three diets in a randomized complete block design with eight pigs per diet. After a 7-d adaptation period, feces and urine were quantitatively collected for 4 days. The diets and feces were analyzed for dry matter, and all samples were analyzed for gross energy (GE). The results indicated that the ATTD of GE was greater (*p* < 0.05) for soybean hulls than for pistachio shell powder, but less (*p* < 0.05) than for corn. Pistachio shell powder provided less (*p* < 0.05) DE than corn (i.e., 1778 kcal/kg vs. 3787 kcal/kg; dry matter basis), but the DE in pistachio shell powder was not different from that in soybean hulls (i.e., 2010 kcal/kg; dry matter basis). In conclusion, pistachio shell powder can be used as an alternative ingredient in diets for growing pigs and provides 1778 kcal/kg of DE on a dry matter basis.

## Simple Summary

Pistachio shells are a co-product of the pistachio industry and are often considered waste. Because pistachio production is increasing, identifying alternative uses for pistachio shells will improve sustainability. In this experiment, the hypothesis that the metabolizable energy in pistachio shell powder is not less than that in soybean hulls when fed to growing pigs was tested. Pigs were fed on a corn-based diet or diets containing pistachio shell powder or soybean hulls. Pistachio shell powder increased fecal output and had lower energy digestibility than corn, which was expected due to its high fiber content. However, the digestible and metabolizable energy in pistachio shell powder was not different from that in soybean hulls when included at 20% of the diet. These results indicate that pistachio shell powder may be used as a fiber ingredient in pig diets.

## 1. Introduction

The global production of pistachios is increasing, and California is the second largest producer of pistachios in the world, with approximately 488,000 planted acres in 2025 [1]. Pistachio nuts are mainly used for human foods because they are a rich source of lipids, protein, and minerals [2]. Pistachio shells are generated as a co-product of nut processing and considered waste. Grinding pistachio shells yields pistachio shell powder, which has been used in diets for ruminants, including lactating cows and goats [3], but data on the nutritional value of pistachio shell powder fed to pigs are limited. Due to the high concentration of fiber, pistachio shell powder may affect energy digestibility and gastrointestinal function in pigs, but knowledge about the digestible energy (DE) and metabolizable energy (ME) is essential for accurate diet formulation with the ingredient. Alternative fiber sources, such as soybean hulls, are sometimes included in pig diets to provide fiber. Comparing pistachio shell powder to soybean hulls may determine if this co-product can serve as a sustainable and cost-effective fiber source in diets for growing pigs. Therefore, we tested the null hypothesis that, for growing pigs, the apparent total tract digestibility (ATTD) of gross energy (GE) and DE and ME in pistachio shell powder is not different from the ATTD of GE and the DE and ME in soybean hulls.

## 2. Materials and Methods

The protocol for the animal part of the experiment was reviewed by the Institutional Animal Care and Use Committee at the University of Illinois before animal work was initiated. A basal diet contained corn was the only source of energy. Two additional diets contained corn and pistachio shell powder or corn and soybean hulls (Table 1 and Table 2). All diets were fortified with vitamins and minerals to meet or exceed current requirement estimates [4]. The same batch of corn was used in all diets.

### 2.1. Diets, Animals, and Experimental Design

Pigs that were used in the experiment originated from Line 800 boars mated to Camborough females (Pig Improvement Company, Hendersonville, TN, USA). Twenty-four growing pigs with an average initial body weight of 32.0 ± 1.7 kg were randomly allotted to the three diets in a randomized complete block design with eight replicate pigs per diet. Initial body weight group was the block. Pigs were housed individually in metabolism crates (0.81 m × 1.52 m). Each metabolism crate had a self-feeder, a nipple waterer, and a slatted floor, and under the metabolism crates, a screen and a urine pan were installed to allow for the total, but separate, collection of urine and fecal materials. Feed was supplied in a daily amount of 3.2 times the maintenance energy requirement (i.e., 197 kcal ME/kg × BW^0.60^; [4]), and pigs had free access to water throughout the experiment.

### 2.2. Sample Collection

Feed consumption was recorded daily. The initial seven days were considered the adaptation period to the diet, whereas urine and fecal materials were collected from the feed provided during the following four days, according to the marker-to-marker approach [5]. Fecal collection was initiated when the first marker (i.e., chromic oxide) appeared in the feces and ceased when the second marker (i.e., ferric oxide) appeared [5]. Urine was collected in urine buckets over a preservative of 50 mL of 6 N HCl. Fecal samples and 10% subsamples of the collected urine were stored at −20 °C immediately after collection. When the animal part of the experiment was completed, urine samples were defrosted and mixed within pig, and a subsample was lyophilized before analysis [6].

### 2.3. Chemical Analysis

Two samples were used for each analysis. Fecal samples were defrosted, dried at 65 °C using a forced air-drying oven, and ground through a 1-mm screen using a grain mill (500 G Swing Type Grain Mill, RRH, Rancho Cucamonga, CA, USA) before analysis. Diets, ingredients, and fecal samples were analyzed for dry matter (DM; method 930.15; [7]), and diets and ingredient samples were also analyzed for dry ash (method 942.05; [7]). Ingredients, diets, fecal, and urine samples were analyzed for GE using bomb calorimetry (Model 6400; Parr Instruments, Moline, IL, USA). Diets and ingredients were analyzed for nitrogen via the combustion procedure (method 990.03; [7]) using a LECO FP628 Nitrogen Analyzer (LECO Corp., Saint Joseph, MI, USA), and crude protein was calculated as analyzed nitrogen × 6.25. Acid-hydrolyzed ether extract was analyzed in ingredients by acid hydrolysis using 3 N HCl (ANKOM HCl, Ankom Technology, Macedon, NY, USA) followed by crude fat extraction using petroleum ether (AnkomXT15, Ankom Technology, Macedon, NY, USA). Insoluble and soluble dietary fiber were analyzed in ingredients according to method 991.43 [7], using the Ankom^TDF^ Dietary Fiber Analyzer (Ankom Technology, Macedon, NY, USA), and total dietary fiber was calculated as the sum of insoluble dietary fiber and soluble dietary fiber. Ingredients were also analyzed for AA [method 982.30 E (a, b, c); [7]] on a Hitachi Amino Acid Analyzer, Model No. L8800 (Hitachi High Technologies America, Inc.; Pleasanton, CA, USA) using ninhydrin for post-column derivatization and nor-leucine as the internal standard.

### 2.4. Calculations and Statistical Analysis

The ATTD of GE and DM and concentrations of DE and ME in all diets were calculated [4,5]. Because corn was the only energy source, the ATTD of GE in the basal diet represented the ATTD of GE in corn. Concentrations of DE and ME in corn were calculated by dividing the DE and ME in the basal diet by the inclusion rate of corn (i.e., 96.47%). The ATTD of GE and values for DE and ME were calculated in pistachio shell powder and soybean hulls by difference [4].

Normality of data was verified, and the UNIVARIATE procedure was used to identify outliers, which were values that deviated from the treatment mean by more than 3 times the interquartile range (SAS 9.4; SAS Inst. Inc., Cary, NC, USA). The pig was the experimental unit for all analyses. Two outliers were removed from the corn diet prior to the calculations of DE and ME in feed ingredients. Data were analyzed using the MIXED procedure of SAS 9.4 (SAS Institute Inc., Cary, NC, USA), with the pig as the experimental unit. Diet was considered the main effect, whereas block was the random effect. The LSMEANS statement and the PDIFF option of PROC MIXED were used to separate means. Statistical significance was considered at *p* < 0.05 and tendency at *p* < 0.10.

## 3. Results

No differences were observed among the diets for daily feed intake, DM intake, or GE intake (Table 3). The weight of dried feces and fecal GE output were greatest (*p* < 0.05) for the pigs fed the pistachio shell powder diet, followed by the soybean hull diet (*p* < 0.05) and the corn diet (*p* < 0.05), but no difference was observed for weight of urine or urine GE output. The ATTD of DM and GE was greatest (*p* < 0.05) for the corn diet, followed the soybean hull diet (*p* < 0.05), and the pistachio shell powder diet had the least (*p* < 0.05) ATTD in terms of DM and GE. The DE and ME in the corn diet were greater (*p* < 0.05) than in the diets containing pistachio shell powder or soybean hulls, but no differences were observed between the pistachio shell powder diet and the soybean hull diet. For the ingredients, the ATTD of GE in corn was greatest (*p* < 0.05), followed by soybean hulls (*p* < 0.05), and pistachio shell powder offered the least (*p* < 0.05) ATTD in terms of GE. Concentrations of DE and ME in corn as-is and on a DM basis were greater (*p* < 0.05) than in pistachio shell powder or soybean hulls, but no differences were observed between concentrations in pistachio shell powder and soybean hulls.

## 4. Discussion

The GE in all diets was as expected when calculated from ingredient analysis, which indicated minimal errors in diet mixing, subsampling, and analysis for GE. The GE in corn and soybean hulls was less than some previous values [4], which may be because both ingredients contained less protein and more fiber than the ingredients used in the past. The analyzed nutrient composition of pistachio shell powder was in close agreement with recently published values [8,9]. The observation that the ATTD of GE and concentrations of DE and ME in corn were greater than in pistachio shell powder and soybean hulls is in agreement with data indicating that starch-rich ingredients are more completely digested than fibrous co-products in growing pigs [4,10]. The ATTD of the GE in soybean hulls was greater than that in pistachio shell powder, which is consistent with the lower concentration of total dietary fiber and the greater proportion of soluble dietary fiber in soybean hulls compared with pistachio shell powder. Soluble fiber is much more fermentable than insoluble fiber, and fermentation in the hindgut contributes volatile fatty acids that can be absorbed and used as energy by pigs [11]. The fermentability of soluble dietary fiber in corn distillers’ dried grains with solubles is almost 90%, whereas insoluble dietary fiber is fermented by only 40% [12]. However, in all cereal grains and cereal grain co-products, there is much more insoluble dietary fiber than soluble dietary fiber [13] and the overall fermentability of fiber, therefore, is low. The insoluble dietary fiber in cereal grain co-products may contribute up to 40 to 50% of the ingredient [14], and overall energy digestibility, therefore, may be low. However, the pistachio shell powder contained almost 80% insoluble dietary fiber and more than 88% total dietary fiber, which is the reason for the large fecal output, reduced ATTD of GE, and low DE and ME in this ingredient.

Co-products from the human food and nut industries are frequently used in pig diets as sources of dietary fiber [15,16]. Almond hulls, pecan shells, and other nut by-products can be included in pig diets without negative effects on growth performance when diets are properly formulated [17,18]. However, pistachio shell powder is chemically distinct from most other nut co-products because of its extremely high concentration of insoluble dietary fiber and very low concentration of soluble carbohydrates and protein. Whereas almond hulls typically contain approximately 40 to 50% total dietary fiber [19], the pistachio shell powder used in the present experiment contained nearly 90% total dietary fiber, which is in agreement with data for other sources of pistachio shell powder [8,9]. A different source of pistachio shell powder was included in diets for sheep without any negative effects on fermentation or growth performance [20]. There are no other feed ingredients that are used in swine diets that contain close to 90% insoluble dietary fiber, but wheat middlings and soybean hulls contain substantial quantities of insoluble dietary fiber, and both of these ingredients may be included in diets for pigs [21,22,23].

Because pigs are ineffective at fermenting dietary fiber compared with ruminants and hindgut fermenting animals, the inclusion of fiber-rich ingredients is usually limited in diets for pigs. In the present experiment we decided to use 20% pistachio shell powder because this level has been successfully used in diets for sows [8]. It was also recently reported that 10% pistachio shell powder can be used in diets for weanling pigs without any negative impacts on growth performance or blood characteristics [9], and it was believed that growing pigs can tolerate more dietary fiber than weanling pigs.

The relatively low levels of DE and ME in pistachio shell powder are primarily a consequence of its fiber composition rather than an indication of analytical or experimental error. Nevertheless, pistachio shell powder provided DE and ME values that were close to the values obtained for soybean hulls when included as 20% of the diet, which indicates that pistachio shell powder may be used as a conventional fibrous ingredient in diets for growing pigs. Indeed, the results of recent research indicated that up to 10% pistachio shell powder can be included in diets for weanling pigs without negatively impacting the growth performance of pigs [9].

An unexpected observation was that ME values were slightly greater than DE values for pistachio shell powder. This result is not biologically plausible because urinary energy losses should always reduce ME relative to DE. However, this discrepancy is a result of known limitations of the difference procedure when ingredients are included at relatively low levels in the diet [5]. Small analytical errors in fecal or urine energy measurements are magnified when back-calculating ingredient values using a lower inclusion rate (i.e., 20%), particularly for ingredients that contribute very little nitrogen. Pistachio shell powder contains very little crude protein and amino acid and, therefore, almost no nitrogen from this ingredient would be excreted in urine. Because urinary energy is primarily derived from nitrogen excretion in the form of urea [24], the contribution of pistachio shell powder to urinary energy was negligible. As a result, small analytical variations in the urinary energy output of the complete diet may have caused the ME values for pistachio shell powder to slightly exceed the DE values, even though true ME is always lower than DE. Therefore, the difference between the DE and ME in pistachio shell powder is believed to be close to zero. It is unlikely that freezing, thawing, and lyophilizing urine samples influenced analyzed energy in the urine because nitrogen is not expected to be impacted by freezing and thawing. In addition, because urine was collected in buckets that contained hydrochloric acid, volatilization of nitrogen was believed to be negligible, and by lyophilizing samples instead of oven drying, the likelihood of volatilization of volatile compounds in the urine was further reduced.

Because pistachio shell powder is high in fiber, the energy is derived almost exclusively from the microbial fermentation of fiber in the hindgut rather than from the enzymatic digestion of other nutrients in the small intestine. Although pistachio shell powder cannot replace ingredients that are high in energy, it can be used in diets where dietary fiber is desired to support gut health or reduce feed cost.

## 5. Conclusions

Pistachio shell powder contains almost 90% total dietary fiber, with the majority being insoluble dietary fiber, which makes this ingredient unique among the ingredients usually added to diets for growing pigs. Because of high insoluble dietary fiber, pistachio shell powder has reduced ATTD in terms of GE and contains less DE and ME compared with corn; however, values are close to those for soybean hulls when fed to growing pigs. Pistachio shell powder may, therefore, be used in diets for growing pigs. The optimum inclusion rate needs to be determined in growth performance experiments to identify the level of pistachio shell powder that can be used without reducing growth performance. The combination of adding fat and pistachio shell powder to diets at the same time also needs to be researched because added fat may eliminate the possible negative effects of reduced diet ME if pistachio shell powder is used.

## Figures and Tables

**Table 1:** Analyzed nutrient composition of ingredients; as-fed basis.

Item, %	Corn	Pistachio Shell Powder	Soybean Hulls
Dry matter	87.49	98.28	91.77
Gross energy, kcal/kg	3817	4702	3733
Crude protein	6.67	6.09	10.17
Acid hydrolyzed ether extract	3.30	1.65	2.83
Ash	1.20	1.12	4.21
Insoluble dietary fiber	11.80	79.80	64.20
Soluble dietary fiber	1.20	8.90	5.10
Total dietary fiber	13.00	88.70	69.30
Indispensable amino acids			
Arg	-	0.43	0.45
His	-	0.10	0.25
Ile	-	0.17	0.38
Leu	-	0.29	0.63
Lys	-	0.24	0.70
Met	-	0.06	0.12
Phe	-	0.20	0.38
Thr	-	0.15	0.35
Trp	-	0.03	0.06
Val	-	0.25	0.47
Dispensable amino acids	-		
Ala	-	0.20	0.42
Asp	-	0.41	0.91
Cys	-	0.09	0.17
Glu	-	0.79	1.09
Gly	-	0.20	0.87
Pro	-	0.21	0.52
Ser	-	0.22	0.51
Lys: crude protein ratio, %	-	3.94	6.88

**Table 2:** Composition (as-fed basis) of experimental diets.

Item	Corn	Pistachio Shell Powder	Soybean Hulls
Ingredients, %			
Ground corn	96.47	76.75	76.75
Pistachio shell powder	-	20.00	-
Soybean hulls	-	-	20.00
Dicalcium phosphate	1.65	1.70	1.70
Ground limestone	0.98	0.65	0.65
Sodium chloride	0.40	0.40	0.40
Vitamin micromineral premix ^1^	0.50	0.50	0.50
Analyzed composition			
Dry matter, %	86.65	88.74	87.14
Gross energy, kcal/kg	3719	3859	3692
Crude protein, %	6.88	6.32	7.05
Ash, %	2.83	3.28	3.46

^1^ The vitamin-micromineral premix provided the following quantities of vitamins and micro minerals per kg of complete diet: vitamin A as retinyl acetate, 10,622 IU; vitamin D_3_ as cholecalciferol, 1660 IU; vitamin E as _DL_-alpha tocopheryl acetate, 66 IU; vitamin K as menadione nicotinamide bisulfate, 1.40 mg; thiamin as thiamine mononitrate, 1.08 mg; riboflavin, 6.49 mg; pyridoxine as pyridoxine hydrochloride, 0.98 mg; vitamin B_12_, 0.03 mg; _D_-pantothenic acid as _D_-calcium pantothenate, 23.2 mg; niacin, 43.4 mg; folic acid, 1.56 mg; biotin, 0.44 mg; Cu, 20 mg as copper chloride; Fe, 123 mg as iron sulfate; I, 1.24 mg as ethylenediamine dihydroiodide; Mn, 59.4 mg as manganese hydroxychloride; Se, 0.27 mg as sodium selenite and selenium yeast; and Zn, 124.7 mg as zinc hydroxychloride.

**Table 3:** Apparent total tract digestibility (ATTD) of dry matter (DM) and gross energy (GE) and concentrations of digestible energy (DE) and metabolizable energy (ME) in experimental diets and ingredients fed to growing pigs ^1^.

Item	Diet			SEM	*p*-Value
	Corn	Pistachio Shell Powder	Soybean Hulls		
Daily feed intake, kg/day	1.32	1.40	1.38	0.07	0.715
DM intake, kg/day	1.15	1.24	1.20	0.06	0.527
GE intake, kcal/day	4868	5404	5083	252	0.318
Weight of feces, kg/day	0.14 ^c^	0.30 ^a^	0.24 ^b^	0.01	<0.001
Weight of urine, kg/day	4.59	3.77	3.35	1.38	0.829
Fecal GE output, kcal/day	641 ^c^	1367 ^a^	1053 ^b^	63	<0.001
Urine GE output, kcal/day	95	64	84	16	0.383
Diet					
ATTD of DM, %	88.4 ^a^	77.0 ^c^	80.6 ^b^	0.68	<0.001
ATTD of GE, %	86.8 ^a^	74.7 ^c^	79.2 ^b^	0.74	<0.001
DE, kcal/kg, as-fed basis	3196 ^a^	2892 ^b^	2914 ^b^	19	<0.001
ME, kcal/kg, as-fed basis	3124 ^a^	2847 ^b^	2850 ^b^	27	<0.001
Ingredient					
ATTD of GE, %	86.8 ^a^	37.2 ^c^	49.4 ^b^	2.64	<0.001
DE, kcal/kg, as-fed basis	3313 ^a^	1747 ^b^	1845 ^b^	118	<0.001
DE, kcal/kg, DM basis	3787 ^a^	1778 ^b^	2010 ^b^	121	<0.001
ME, kcal/kg, as-fed basis	3239 ^a^	1807 ^b^	1824 ^b^	109	<0.001
ME, kcal/kg, DM basis	3702 ^a^	1838 ^b^	1988 ^b^	114	<0.001

^a–c^ Means within a row without a common superscript differ (*p* < 0.05). ^1^ Least square means represent 6 replicates for corn and soybean hulls and 8 replicates for pistachio shell powder.

## Data Availability

The majority of the data supporting the conclusions of this manuscript are provided within the manuscript.
